# Intermediary Management and Employee Corporate Culture Identification Mediation and Mediation Effect Verification

**DOI:** 10.3389/fpsyg.2021.545816

**Published:** 2021-10-13

**Authors:** Mingji Liu, Jinyao Li, Tianlang Xiong, Tong Liu, Min Chen

**Affiliations:** ^1^School of Economics and Management, Harbin University of Science and Technology, Harbin, China; ^2^College of Public Administration, Shandong Technology and Business University, Yantai, China; ^3^College of Economics, Sichuan Agricultural University, Chengdu, China; ^4^College of Humanities and Society, Jeonju University, Jeonju, South Korea; ^5^College of Business, Zhejiang University, Hanzhou, China

**Keywords:** intermediary management, employee corporate culture, identification mediation, mediation effect verification, employee involvement

## Abstract

This exploration is mainly performed to study the role of corporate culture accepted by employees in enterprise development and its impact on employees themselves. First, the influence of employee participation, cross-cultural management, and corporate culture on the enterprise is realized through the relevant literature. Then, investigation and analysis are carried out with American I Industrial Group as the research object to determine the impact of cross-cultural management on mergers and acquisitions and organizational performance. The results show that the total impact of trust on reuse is 0.264 before mergers and acquisitions; the difference is not statistically significant, and so is the overall impact of mergers and acquisitions. This means that there is no correlation between trust and reuse. However, when the merger is done, the total effect of trust on reuse rises to 1.594, indicating that the difference and the total effect are statistically significant. The data calculation and analysis for the direct impact of trust on reuse and the indirect impact of trust on reuse are 0.667 and 0.926, respectively, which means that the difference is statistically significant. This proves the role of satisfaction in the impact of trust on reuse once mergers and acquisitions are completed. Therefore, in the process of mergers and acquisitions in the future, enterprises must consider the different cultures of employees and company locations and employee participation, which will further affect the organizational performance of enterprises.

## Introduction

The theory of corporate culture was born in the early 1980s. More and more people have realized the power of corporate culture and the inestimable role of culture in the development of enterprises with the popularization of corporate culture theory. Chinese enterprises face increasing competitive pressure with the accelerating process of world economic integration. How to make Chinese enterprises comply with the trend of market development and how to create the maximum value of enterprises have become major issues that every enterprise, especially state-owned ones, must take seriously and should be included in the agenda in time. Corporate culture is a value belief and code of conduct gradually cultivated in the process of continuous progress and improvement, which can be recognized and followed by employees. Corporate culture suitable for enterprise development is very important for every enterprise. Excellent foreign and domestic enterprises both have one thing in common, and that is they all pay great attention to the construction of corporate cultures, such as Haier and Huawei. Enterprises that do not pay enough attention to the construction of corporate culture usually find it difficult to maintain good development. The corporate culture construction in China has started late, the overall level is still relatively low, and there is still a long way to go (Akiate, 2018). At present, the cultural construction of many domestic enterprises still stays on the surface and is not really implemented, and the real connotation of corporate culture construction is often ignored. They believe that corporate culture is to organize some superficial activities. More seriously, so far, the corporate culture construction of some companies is only a slogan and a dead letter, and its pace has stagnated.

Since reform and opening up, the economy of China has gradually entered an era of rapid development, but Chinese enterprises are also facing unprecedented competitive pressure at the same time. Nowadays, the core of the world economy and the competition among enterprises have gradually changed into the competition of cultural soft power. Corporate culture plays an increasingly crucial role in the development of an enterprise (Sosnilo and Snetkova, 2020). Since the birth of corporate culture theory, the construction of corporate culture has been gradually attracting the attention of Chinese business circles, academia, and even government departments. Domestic enterprises have begun to explore the road of corporate culture construction, and some excellent enterprises have established a relatively perfect corporate culture system suitable for their development, but the cultural construction of many enterprises still stays on the surface and needs continuous exploration and research to move forward (Faizaty et al., 2020). It can be said that building an excellent corporate culture is a problem that every enterprise, especially state-owned ones, should think about and solve.

Therefore, the role of the corporate culture of employees in the enterprise is investigated and analyzed. First, the influence of employee participation, cross-cultural management, and corporate culture on the enterprise is realized through relevant literature. Then, investigation and analysis are carried out with American I Industrial Group as the research object to determine the impact of cross-cultural management on mergers and acquisitions and organizational performance. The innovation of this study is to use cross-cultural management as an intermediary to study and verify the intermediary relationship between intermediary management and employees' corporate culture identity. In this process, the relationship among employees of multinational corporations is regarded as a measure of different degrees of cross-cultural identity in the context of cross-cultural management.

## Literature Review

With the increasing level of economic globalization at this stage, massive enterprises have begun to pursue the internal cultural construction of enterprises to seek high-quality development and stimulate the desire to work and efficiency of employees by their cultural construction. Therefore, many scholars have begun to focus on corporate culture.

Aisyah et al. (2020) found that organizational culture variables had a significantly positive impact on employee performance, and those incentive variables also had a significant and positive impact on the performance of employees. Cultural variables and motivation, together, had a positive and significant impact on employee performance. Svistunov et al. (2021) explored the relationship between employee satisfaction and the digital level of a company and found that job satisfaction was considered to be an important factor in the formation and development of corporate culture. Meanwhile, with the increasing use of modern information technology tools, the creativity and incentive attitude of employees were reduced. In a digital context, formulating an effective strategy for the interaction between the top management of a company and its employees mainly depends on the automation level of business processes in the corporate culture of the company and the satisfaction of employees with working conditions. Al-Louzi (2019) found that leadership style, corporate culture, authorization, goal clarity, employee training, employee motivation, and information technology play an important role in the innovation of a management model. Therefore, it is necessary to cultivate an organizational environment that supports creativity based on a flexible organizational culture, so that employees could have an opportunity to participate in decision-making, solve problems, delegate power, simplify work production, and promote innovative thinking. Frolova and Mahmood (2019) found that local and multinational companies had a high level of employee responsibility orientation and that there were differences in employee responsibility orientation. Among different personality traits, responsibility had the greatest positive impact on responsibility orientation, while emotion had the greatest negative impact on responsibility orientation. In different leadership styles, autocratic leadership had the most significant impact on responsibility orientation. Among the types of organizational culture, political culture had the most significant positive impact on responsibility orientation. Urbancová and Depoo (2021) studied the impact of the type of organizational culture on the implementation of human resource activities and employer brand. They found that choosing the appropriate type of organizational culture helped in successfully building an employer brand and work investment, and that brand recognition and communication directly improved the positive perception of organizational culture. Trofimov et al. (2019) found that loyalty level, attitude, and labor values of enterprise employees largely determined the external (salary, welfare, and working conditions) and internal (work content, professional growth opportunities, recognition and performance evaluation) incentive sensitivity. Almeida and Coelho (2019) studied the impact of corporate culture and communication between managers and employees on corporate reputation and image, as well as their impact on the attitudes and behaviors of employees. They found that the cognition of corporate social responsibility practice could improve the cognition of corporate social responsibility and enhance the relationship between employees and organizations. As companies become more involved in social responsibility practices, corporate reputation, commitment, and productivity may be strengthened.

The above experts' and scholars' research on the impact of corporate culture on enterprise employees has provided help for enterprise management to a great extent, and provided research ideas for the role of corporate culture in the enterprise. Therefore, corporate culture will become a key factor to improve employees' work enthusiasm and promote enterprise development in the future.

## Methodology

Cultural differences are the greatest stumbling block to the foreign investment of multinational companies as far as the management performance of international operators is concerned (Alserhan and Shbail, 2020). Since organizational stakeholders in different companies have cultural communication gaps, managing cross-cultural consensus is the responsibility of managers (Kussin and Bundtzen, 2021). If the first task of performance fails to reach a consensus, this will negatively affect the effectiveness of mergers and acquisition integration and, hence, business performance (Ili, 2020). When conducting mergers and acquisitions, the principal corporation considers its organizational culture and operating management system to be superior to those of the subsidiary. Thus, consolidation can only be enhanced through the adoption of the operation management system of the principal corporation. Farooq et al. (2019) suggested that a selection of differing cross-cultural management styles involving the change of management and welfare system, changing staff morale, and different business performance is the reason why cross-cultural management in multinational corporations is faced by corporate identity challenges for its employees following the transition (Farooq et al., 2019). Based on this, this study has developed a research hypothesis aimed at verifying the mediating effects of cross-cultural management and multinational corporate employee cultural identity based on the relationship among the variables (Najib and Nawangsari, 2021).

### Research Subject: American I Industrial Group

I companies are multinational corporations in the United States operating in 57 different countries and employing over 50,000 professional employees, and they have more than 17,000 authorized and pending patent portfolios (Rodríguez-Sánchez et al., 2018). This study also focuses on 359 internal personnel of the welding business group in the Greater China Region and 14 Southeast Asian companies in the six corporate global business groups (Kucharska and Kowalczyk, 2019). The 14 companies experienced different cultural patterns before and after the acquisition. The functions of the research object include production, operations, marketing, sales, and other fields while the period of study was 5 years, from 2012 to 2017.

### Cross-Cultural Management Intermediary Variations: Toolbox Management Method of I Enterprises

Based on the differences in the cross-cultural management model, a 5-year study on I after the acquisition of the business management model found that the corporation adopted the cultural management model using the second model after the acquisition, gradually transitioning to the first three models before finally reaching the first mode (Uzyumova, 2019). In order to reflect the goal of management research on the alienation of the I company, this study uses the management toolbox principle of the I company (Kazi and Chandani, 2021) as a cross-cultural management tool and cultural intermediary variable to express the significance of corporate values and culture (Song et al., 2020). To design the vouchers, Odukoya et al. (2020) suggested that the questionnaire use the level of recognition and use the toolbox to represent the degree of corporate cultural identities of employees in cross-cultural management and to conduct hypotheses of mediating effects. This study also considers the hypothesis of the adjustment effect of joining the personnel's entry time and management position according to the timing of corporate acquisition (Smerek and Vetráková, 2020).

The toolbox is a set of practical business processes developed by I companies over the years, and the main tool for disseminating its core culture among many different business units and regions (Shaheer et al., 2020). Its content is covered by: 80/20 - major analytical tools and products; line simplification (PLS); in-lining, market demand rate (MRD); understanding, simplification, and action (USa); market segment (MSS). Their respective areas of corporate sector coverage and practical implications are briefly described as follows:

#### The Main Working Principle (80/20)

I companies use the 80/20 principle in transnational and cross-cultural management to analyze the data collected by each business entity in the business. By implementing this process, each business person must focus on the 20% of customers who can earn 80% of their revenue and provide services around these key customers (Schwens et al., 2018).

#### Product (PLS)

Simplify product offerings and consider facing coverage: engineering and manufacturing capabilities, marketing strategies, product fit for business and return, target customer needs (Dastgeer et al., 2020).

#### Production (In-lining)

In a simple and consistent production process, related matters include manufacturing, production equipment location, and assembly station improvement, reducing original processing time, and material to finished product single-line management (Tran and Pham, 2019).

#### Warehousing (Market Demand Rate, MRD)

Drive system supply according to customer demand and use rate to supplement specific inventory targets to meet customer needs.

#### Operation (USa)

USa's tool for streamlining workflows: U (understand) refers to uncovering/discovering/locating/identifying non-value-added steps in the process, then allocating costs, S (simplified) refers to the assumption or simulation redesigned and simplified workflow, a (behavior) refers to planning and implementing a new job flow (Wang et al., 2020). It is mainly used to pay suppliers, shut down production lines, reduce warranty claim costs, reduce inventory, etc.

#### Marketing Segmenting (Market Segment Selling)

Focus on sales targets and activities to provide higher sales and profits and increase market share (Svistunov et al., 2021). Its actions include collecting data (end users, competition, government, industry, etc.) to understand the needs and value of the end market, using data to formulate solid marketing strategies, establishing sales strategies and action plans, and effectively implementing plans, and measuring results (Zhou et al., 2019).

### Research Framework and Assumptions

**Figure 1:**
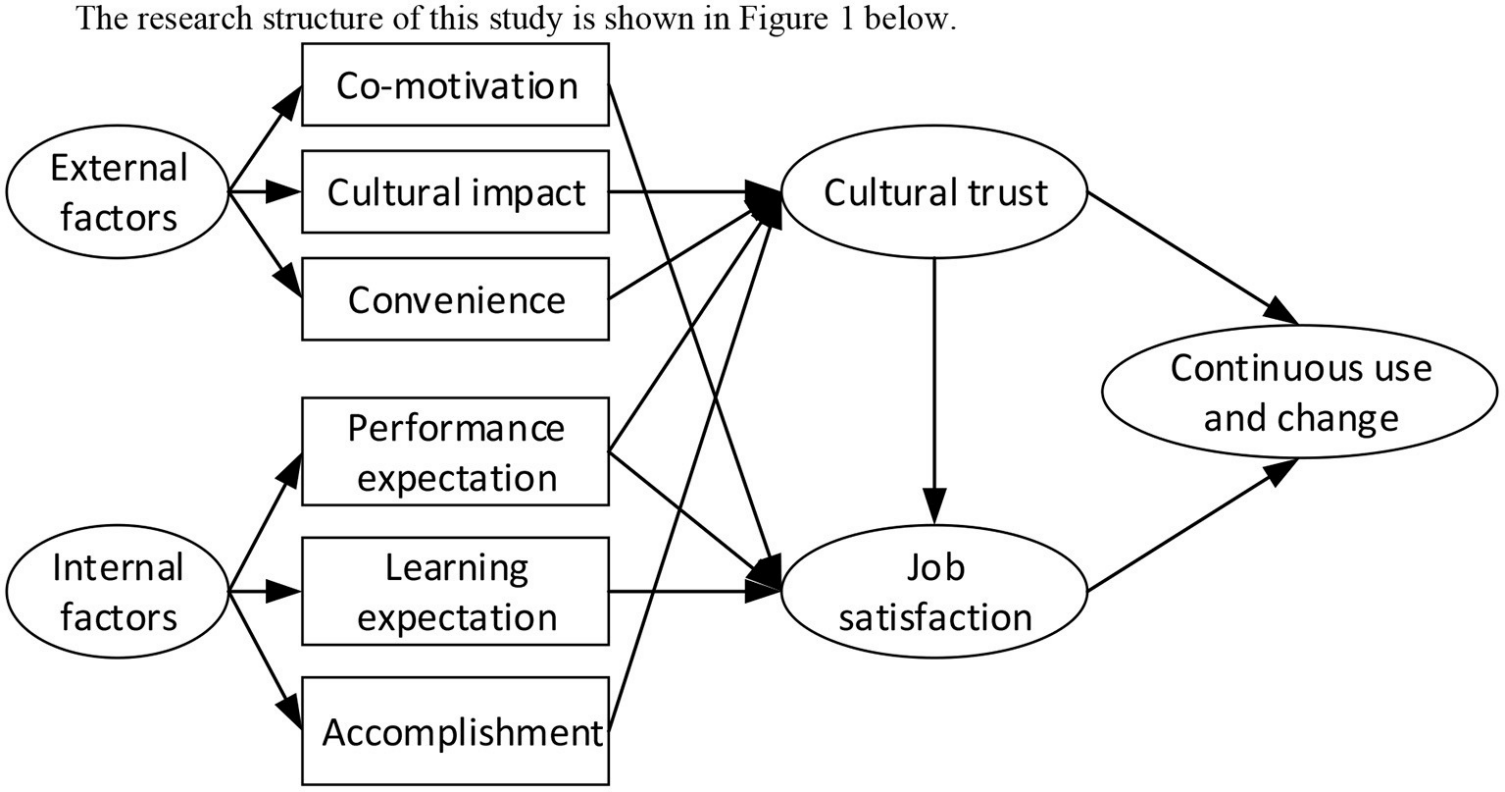
Analysis of requirements.

The research structure of this study is shown in Figure 1.

Based on the above document collation and research framework, the assumptions made in this study are summarized and shown in Table 1.

**Table 1:** Research hypothesis.

**Variable**		**Hypothesis**
Internal hypothesis	H1	The more co-workers motivate (use toolbox), the more they achieve job satisfaction.
	H2	Higher corporate culture (toolbox) influence leads to increased trust in the corporate culture.
	H3	Higher ease of use leads to increased trust in corporate culture.
	H4	Higher degree of cultural trust of the corporation (toolbox) leads to more willingness to use and change the timing.
External hypothesis	H5	(Using toolbox) Higher performance expectation leads to higher cultural trust and job satisfaction.
	H6	The higher the learning (toolbox) expectation, the higher the job satisfaction.
	H7	Higher sense of accomplishment (using the toolbox) leads to increased job satisfaction.
	H8	(Using toolbox) Higher job satisfaction leads to increased willingness to continue to use the change.
	H9	Higher degree of corporate cultural trust (toolbox trust) leads to higher job satisfaction.
Moderator: internal and external control	H10	The degree of trust in management culture (toolbox trust level) is stronger than the non-management level.
	H11	The degree of cultural trust (toolbox trust) of employees entering the corporation after the acquisition is stronger than that of the corporate employees.
	H12	Non-managerial job satisfaction (using toolbox) is higher than managerial job satisfaction.
	H13	After entering the corporation, the employee's satisfaction with the work (using the toolbox) is stronger than that of the corporate employee
	H14	Management staff and employees entering into the corporate culture are more willing to continue using and changing (using toolbox)

The bootstrap method is adopted for statistical inference. In this method, the statistics obtained by multiple random sampling (usually no <1,000 times) are used to replace the statistics of the overall sample. This method has two advantages: the assumption of independent and identically distributed and limited samples. A new statistic is constructed by repeated sampling to test the results of the regression. For each resampling, regression is performed to calculate the timing coefficient and the corresponding t-statistic under the sample. This process is repeated 2,000 times to obtain the distribution of the new t-statistic. Then, the *p*-value is calculated as


(1) $$\begin{array}{l} P=\frac{1}{N}\overset{N}{\underset{n=1}{\sum }}I_{t_{1}^{n}>t_{0}} \end{array}$$

*N* represents the number of random sampling, $t_{1}^{n}$ represents the t value of regression coefficient after each random sampling, and t_0_ represents the t value of timing coefficient of original estimation. $I_{t_{1}^{n}>t_{0}}$ is a dummy variable. It is 1 when the t value of the regression coefficient of random sampling is greater than the *t* value of the timing coefficient of the original estimation. Otherwise, it is taken as 0.

## Data Analysis and Research Findings

The study examined the behavior of 359 internal personnel of the Welding Business Group in Greater China and Southeast Asia, which is one of the six global business groups of American Transnational Industrial Group I, as the main research objects (Farooq et al., 2019). The corporation has 14 departments and employs 17 foreign senior managers, 28 Chinese middle managers, and 314 ordinary employees. Foreign employees account for 7% of the total number of employees, and there are 45 post-managers, accounting for 12.5% of the total number of employees (Romani et al., 2018). The study employed various tools such as physical questionnaires, electronic questionnaires, Internet access, and interviews in conducting anonymous data collection and model analysis (Phan et al., 2020). The research analysis process and research findings are outlined below.

### Confirmatory Factor Analysis

**Figure 2:**
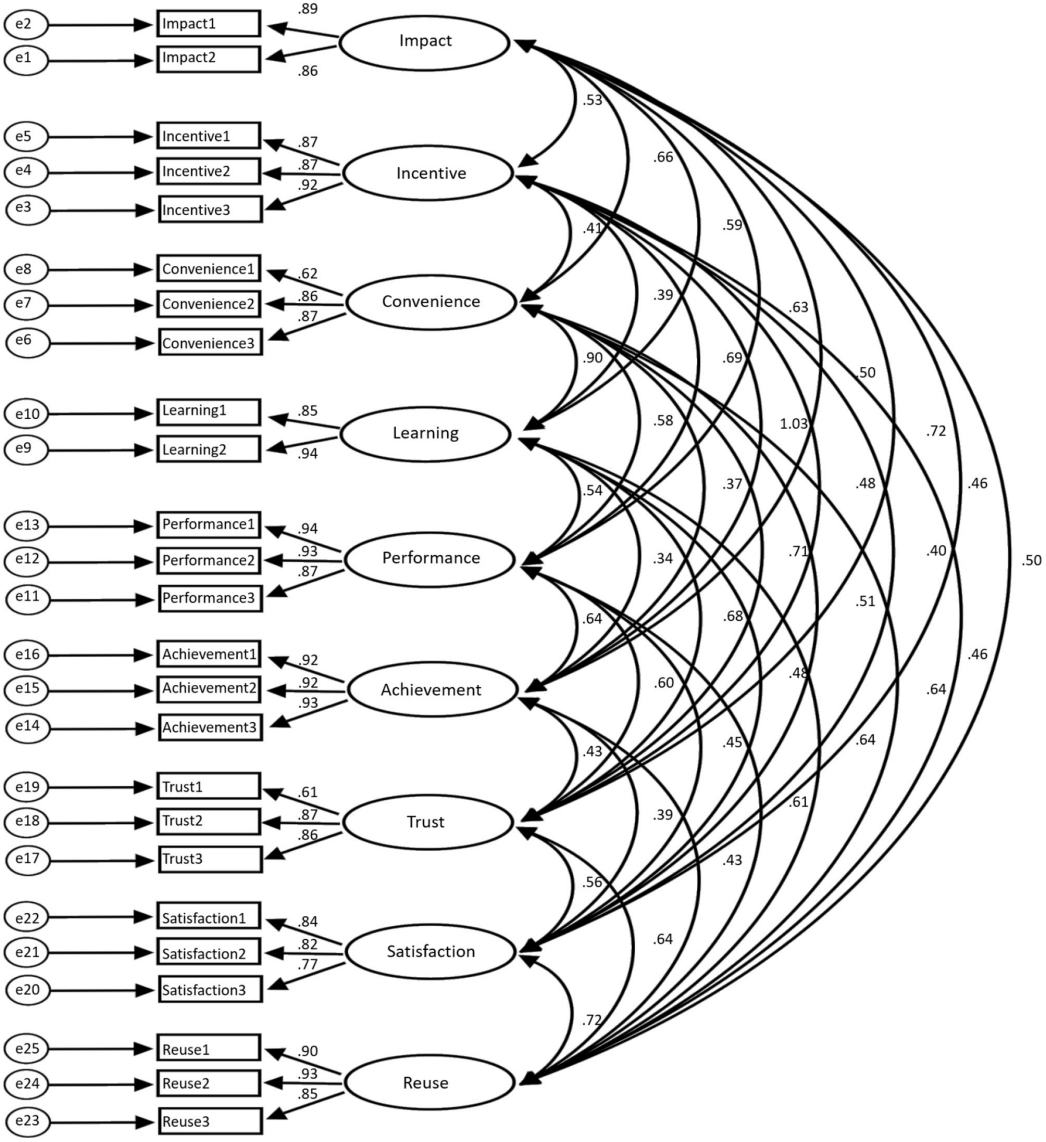
Confirmatory factor analysis model.

**Figure 3:**
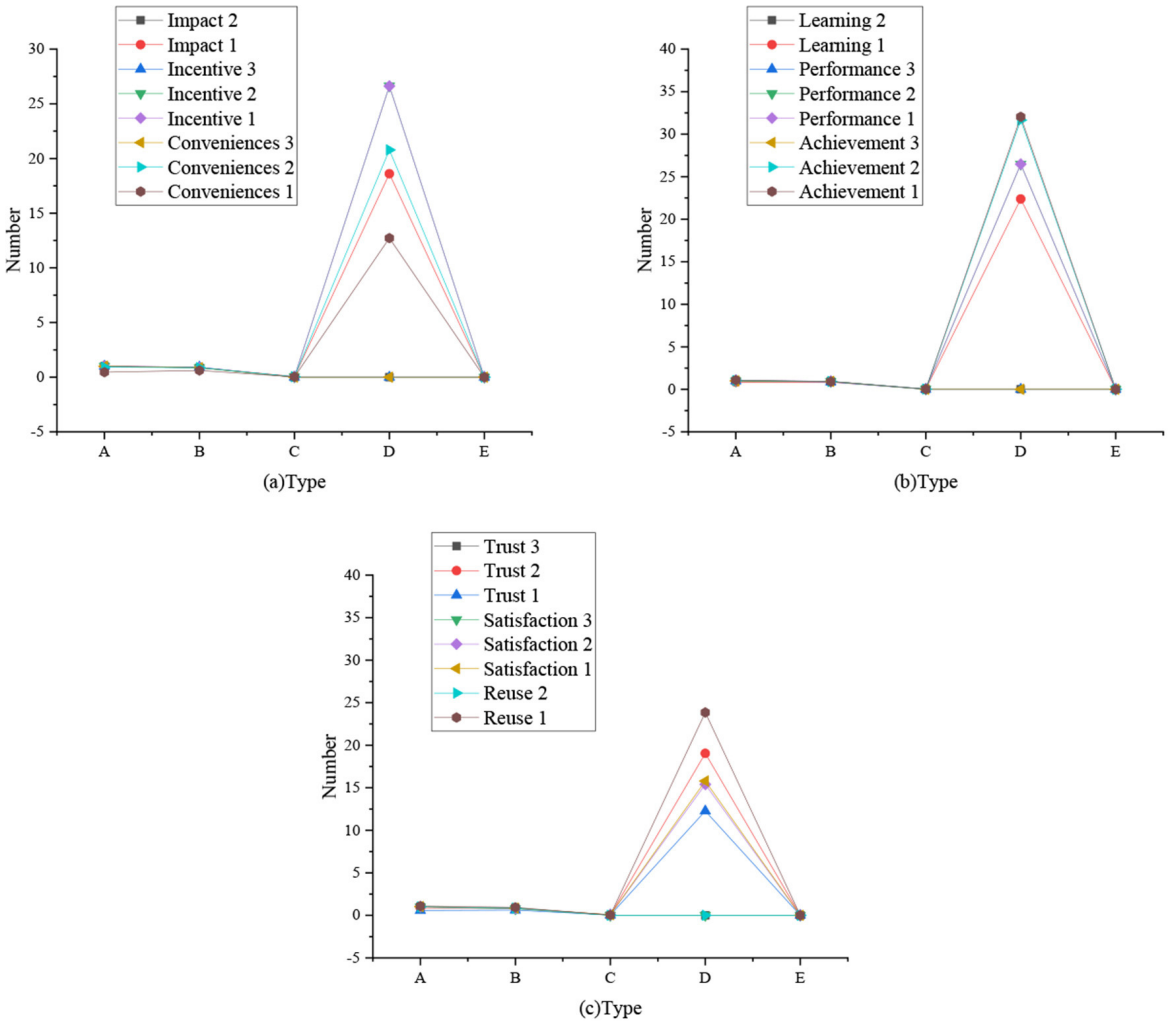
Regression weight statistics of various factors (**a**: regression weight of influencing, incentive, and convenience factors; **b**: regression weight of learning, performance, and achievement factors; **c**: regression weight of trust, satisfaction, and reuse factors; A: non-standard; B: standardized C: SE value; D: *T*-value; E: *P*-value).

Figure 2 shows a confirmatory factor analysis model based on the dimension of the questionnaires. Figure 3 shows the regression weight statistics of various factors, and Table 2 shows the summary of model fitting.

**Table 2:** Model fit summary.

**Fit**	**χ^**2**^**	**df**	**χ^**2**^/df**	**RMSEA**	**GFI**	**PCFI**	**CFI**	**NFI**	**IFI**
Model	1064.501	239	4.454	0.078	0.908	0.723	0.907	0.902	0.908
Criteria			<5	<0.08	>0.9	>0.5	>0.9	>0.9	>0.9

The data statistics in Figures 2, 3 show that the proposed theory and the first-order nine-factor confirmatory factor analysis model have identification convergence. The non-standardized estimation has no negative error variance, indicating that it does not violate the model recognition rules. The fitting results conform to the standard, so the questionnaire has good validity and can fully meet the survey and research.

### Mediation Analysis

**Figure 4:**
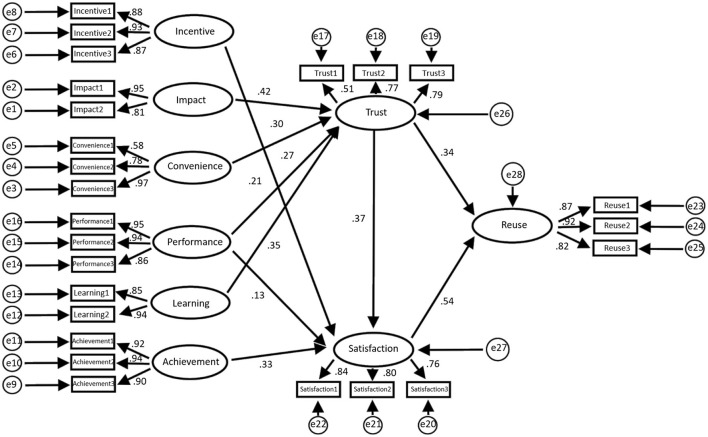
Equation model of the construction agency regression weight.

**Figure 5:**
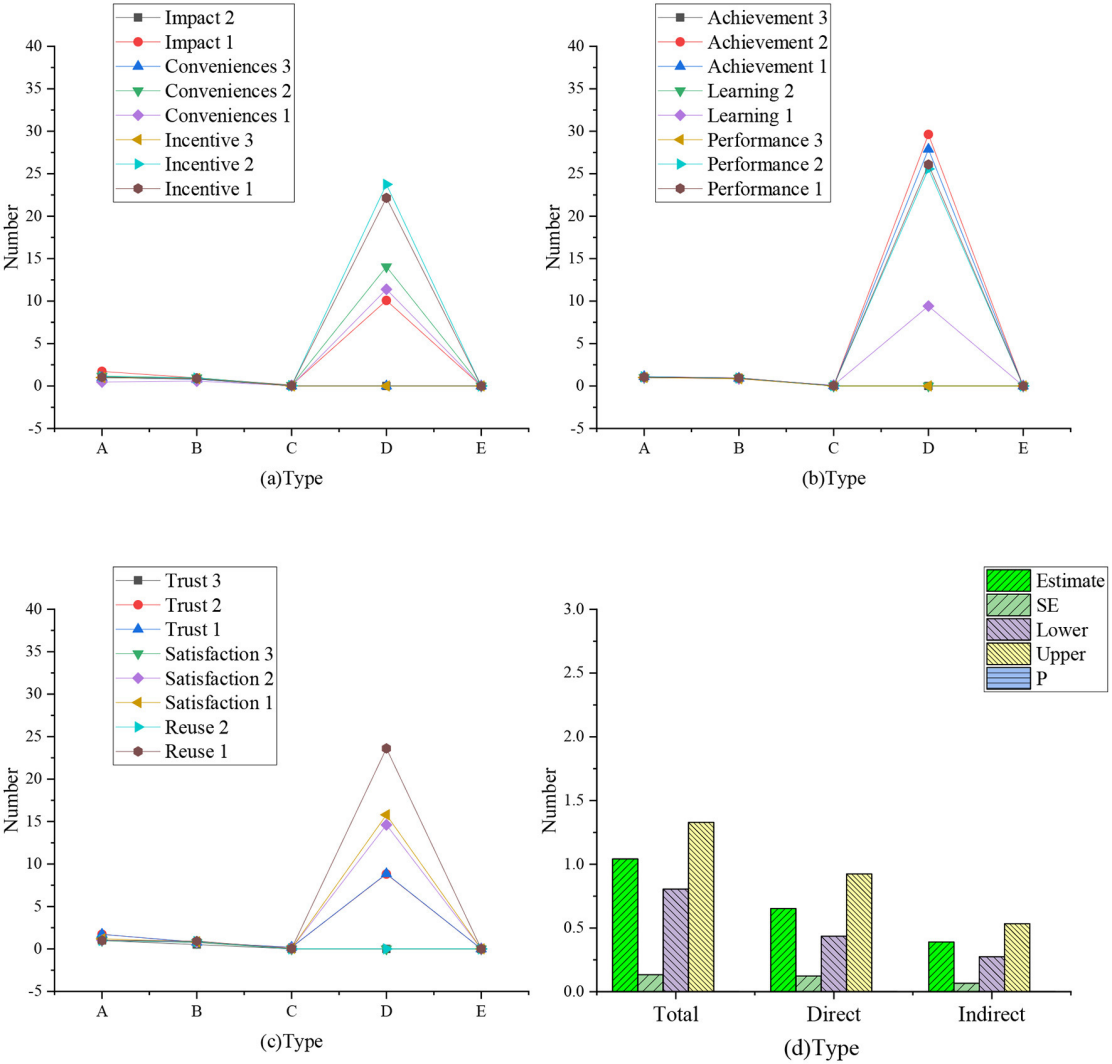
Employee regression weight (**a**: regression weight of influencing, incentive, and convenience factors; **b**: regression weight of learning, performance, and achievement factors; **c**: regression weight of trust, satisfaction, and reuse factors; **d**: mediation analysis conducted by employees; A: non-standard; B: standardized C: SE value; D: *T*-value; E: *P*-value).

Figure 4 shows the structural equation model based on this research framework, and Figure 5 displays the data statistics of the model.

The data statistics in Figure 5 show that the total impact of trust on reuse is 1.042, *P* = 0.001 ≤ 0.05, and that the difference is statistically significant; therefore, the total effect is considered to be significant. Similarly, the direct and indirect impacts of trust on reuse are also significant. This means that t satisfaction plays a mediating role in the impact of trust on reuse, of which mediation accounts for 37.3%.

### Mediation Analysis With Regulation

#### Comparison Between Managers and Non-management Personnel

**Figure 6:**
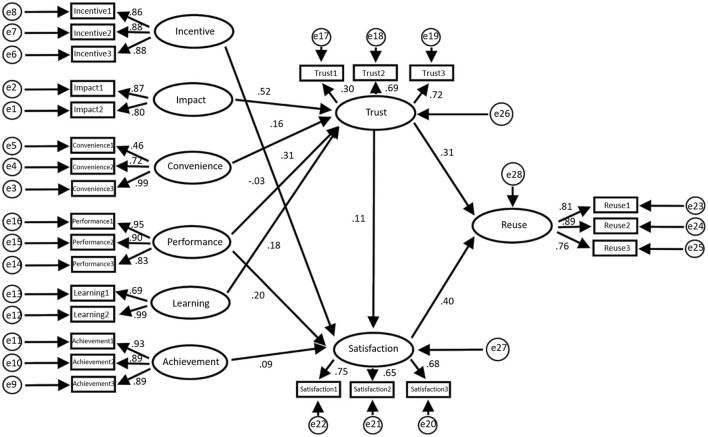
Employee model.

**Figure 7:**
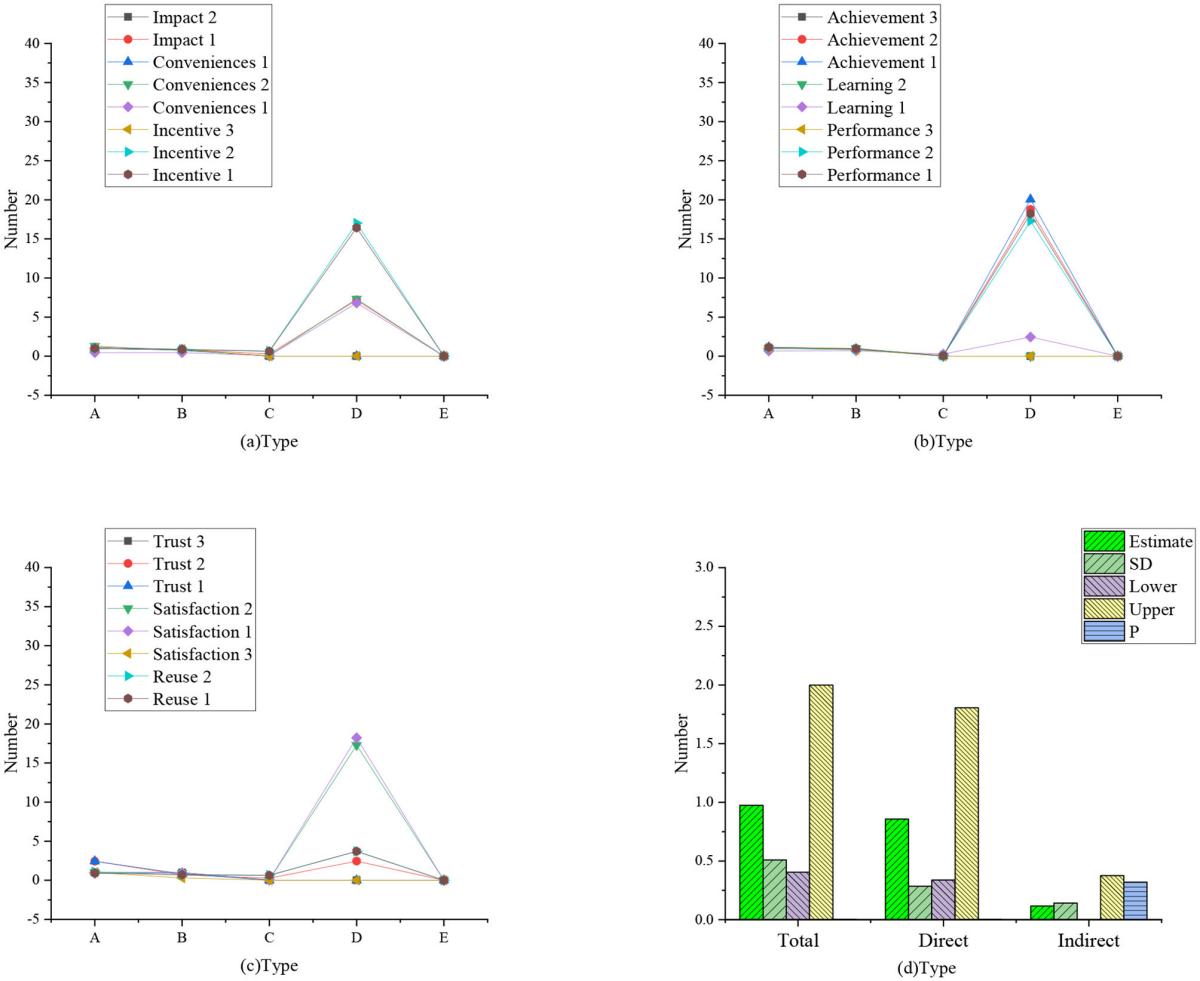
Employee regression weight (**a**: regression weight of influencing, incentive, and convenience factors; **b**: regression weight of learning, performance, and achievement factors; **c**: regression weight of trust, satisfaction, and reuse factors; **d**: mediation analysis conducted by enterprise administrator; A: non-standard; B: standardized C: SE value; D: *T*-value; E: *P*-value).

**Figure 8:**
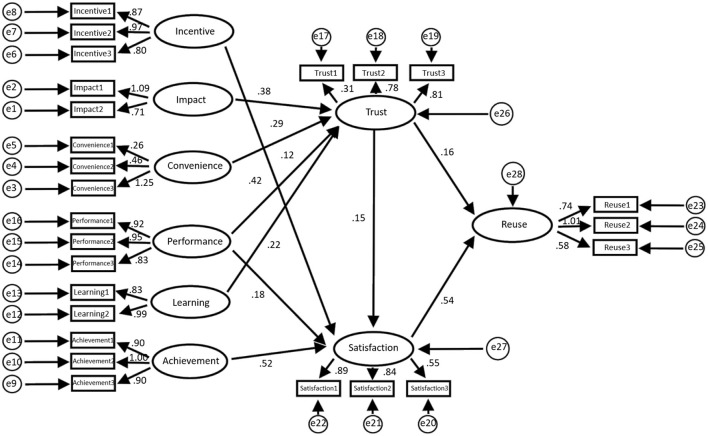
Administrator model.

**Figure 9:**
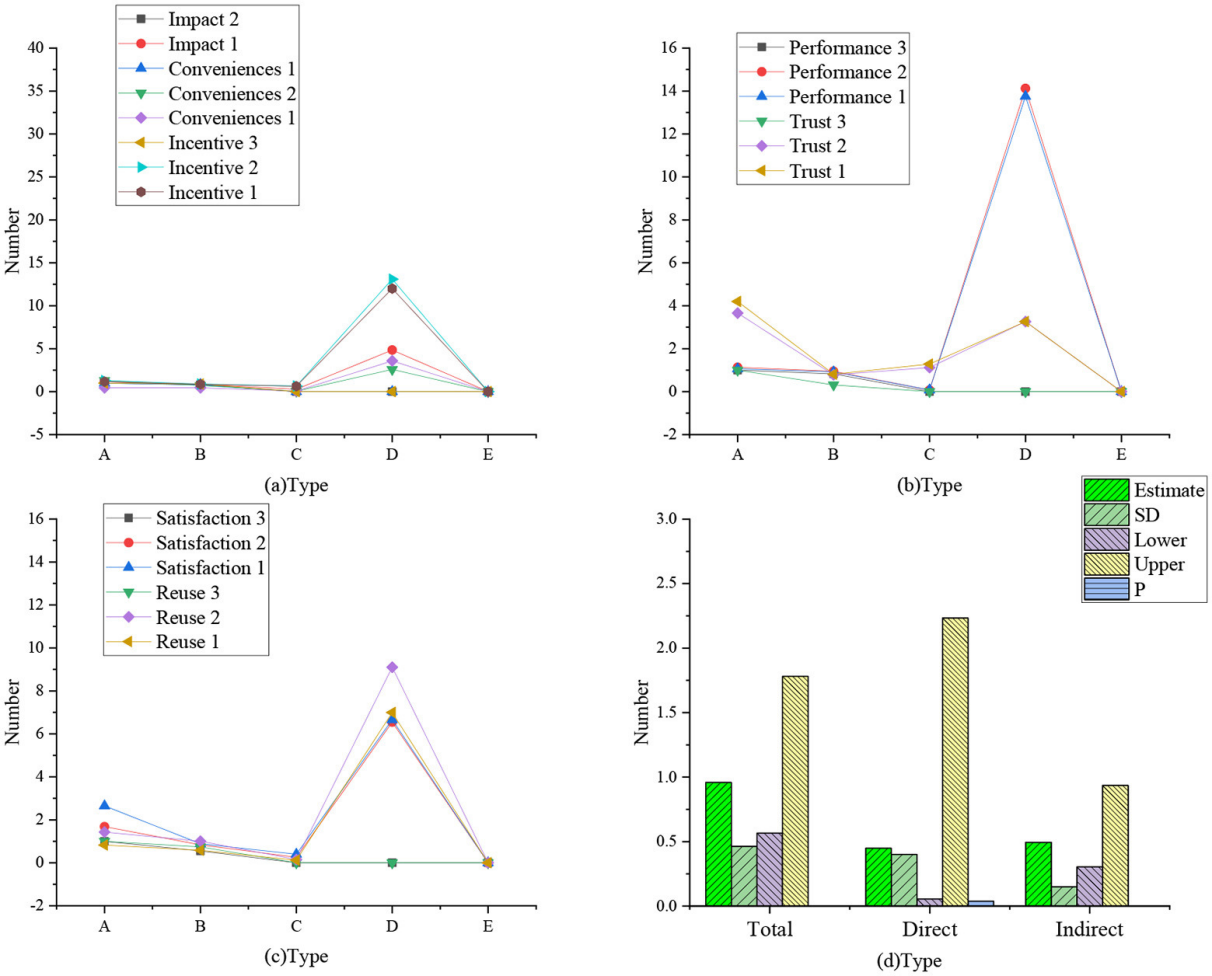
Administrator regression weight statistics (**a**: regression weight of influencing, incentive, and convenience factors; **b**: regression weight of learning, performance, and achievement factors; **c**: regression weight of trust, satisfaction, and reuse factors; **d**: mediation analysis conducted by enterprise administrator; A: non-standard; B: standardized C: SE value; D: *T*-value; E: *P*-value).

The study explored whether there are differences between the characteristics of managers and those of non-management personnel, using multiple groups of analysis based on the mediation effect theory. The results of the analysis are presented in Figures 6, 7. Figures 8, 9 present the data model structure of the administrator and regression weight statistics, respectively.

The data statistics in Figure 7d show that for employees, the total impact of trust on reuse is 0.974, *P* = 0.002 ≤ 0.05. Therefore, the difference is statistically significant, but the total effect is considered to be significant. The indirect impact of trust on reuse is 0.117, *P* = 0.32 > 0.05; therefore, the difference is not statistically significant, indicating that the indirect impact of trust on reuse (mediation effect) is not significant. Therefore, for employees, satisfaction has no mediating effect on the impact of trust on reuse.

The data statistics in Figure 9 show that the total impact of trust on reuse is 0.958, *P* = 0 ≤ 0.05; therefore, the difference is statistically significant, but the total effect is considered to be significant. On the other hand, the indirect impact of trust on reuse is 0.495, *P* = 0.000 ≤ 0.05; therefore, the difference is statistically significant. The direct impact of trust on reuse is 0.449, *P* = 0 ≤ 0.05; therefore, the difference is statistically significant. Hence, for administrators, satisfaction plays a partial mediating role in the impact of trust on reuse.

#### Comparison Before and After M and As

**Figure 10:**
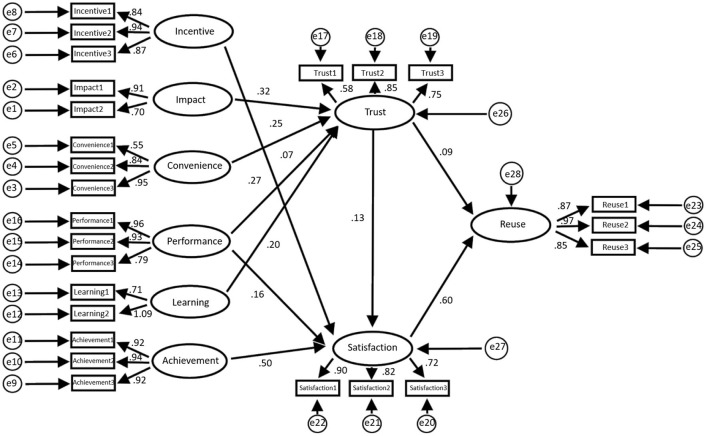
Before model.

**Figure 11:**
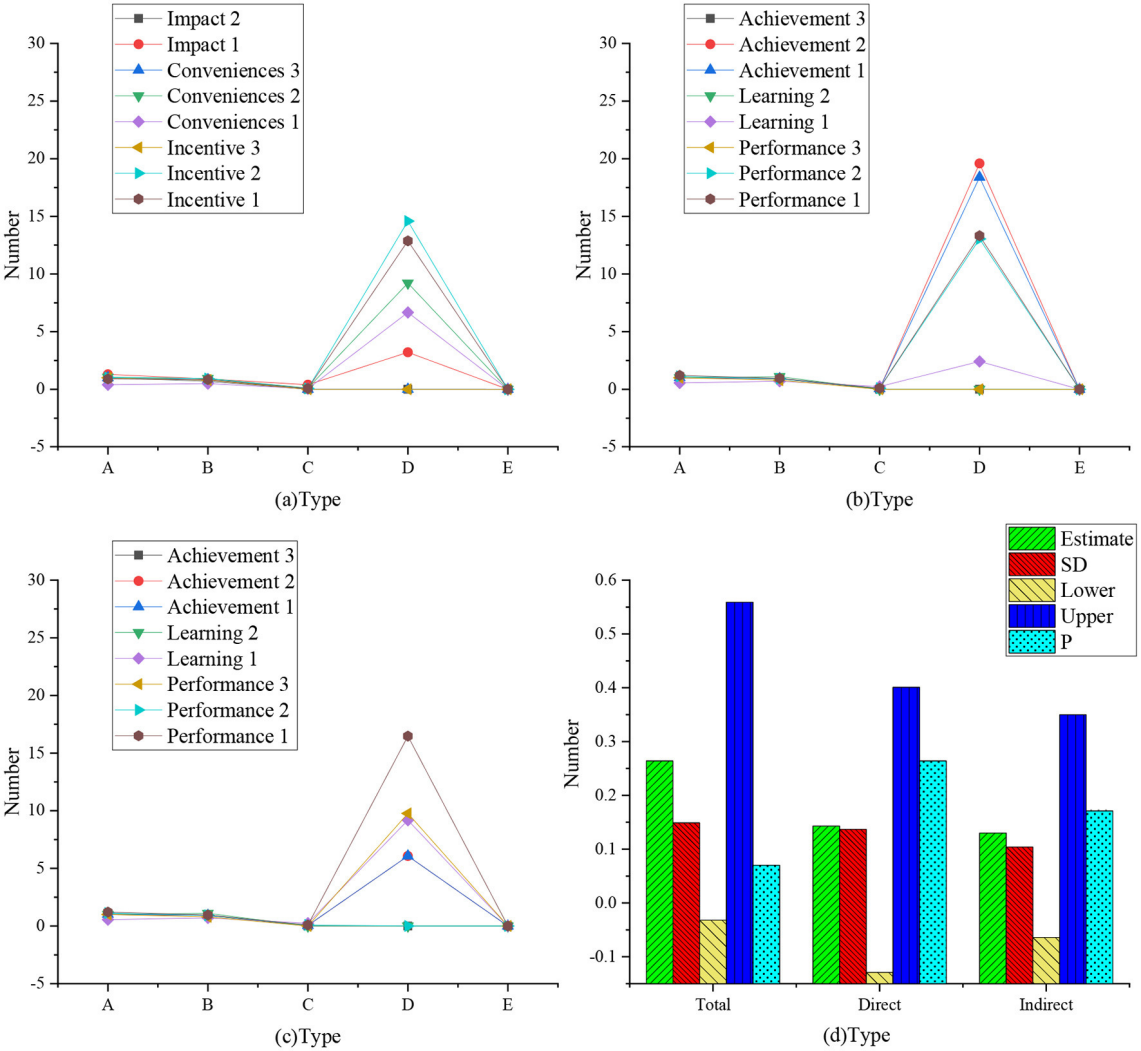
Regression weights before the merger (**a**: regression weight of influencing, incentive, and convenience factors; **b**: regression weight of learning, performance, and achievement factors; **c**: regression weight of trust, satisfaction, and reuse factors; **d**: arbitration analysis before the merger; A: non-standard; B: standardized C: SE value; D: *T*-value; E: *P*-value).

**Figure 12:**
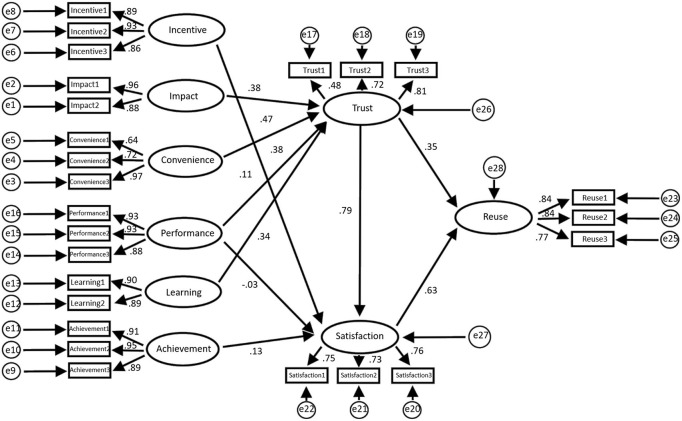
After model.

**Figure 13:**
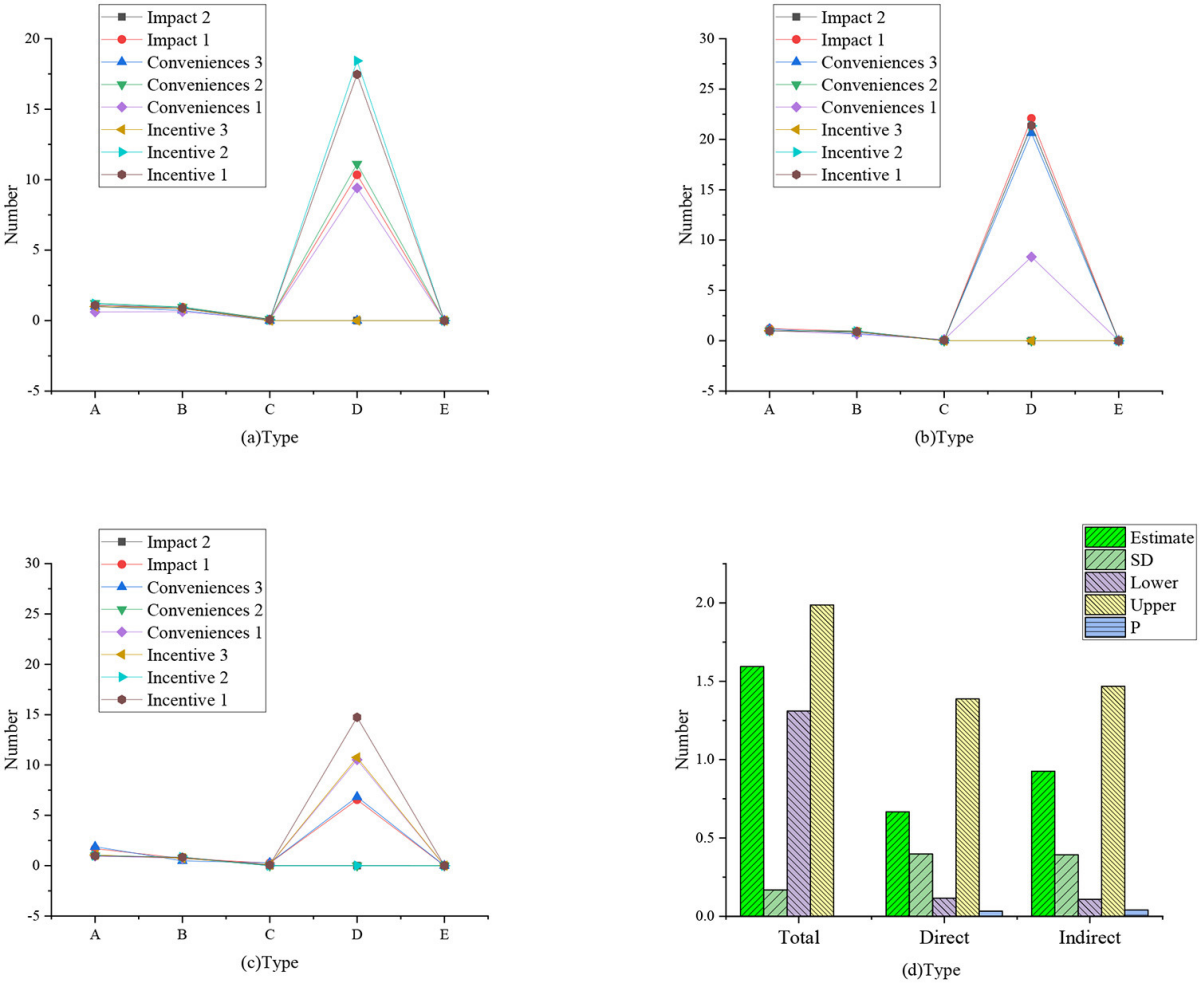
Regression weights after the merger (**a**: regression weight of influencing, incentive, and convenience factors; **b**: regression weights of learning, performance, and achievement factors; **c**: regression weight of trust, satisfaction, and reuse factors; **d**: arbitration analysis after the merger; A: non-standard; B: standardized C: SE value; D: *T*-value; E: *P*-value).

Figures 10, 11 show the model structure and regression weight before the merger, respectively, while Figures 12, 13 show the model structure and regression weight after the merger, respectively.

The data statistics shown in Figure 11 suggest that the total impact of trust on job satisfaction is 0.943, *P* = 0 ≤ 0.05; therefore, the difference is statistically significant, and the total effect is considered to be significant. Besides, the indirect impact of job satisfaction on trust is 0.432, *P* = 0 ≤ 0.05; therefore, the difference is statistically significant. The direct impact of job satisfaction on reuse is 0.432, *P* = 0 ≤ 0.05; therefore, the difference is statistically significant. Hence, for managers, job satisfaction plays a mediating role in the impact of trust on reuse.

The data statistics in Figure 13 clearly show that before the merger, the total impact of trust on reuse is 0.264, *P* = 0.07 > 0.05; therefore, the difference is not statistically significant, and the overall impact is not significant. Hence, there is no correlation between trust and reuse. After the merger, the total impact of trust on reuse is 1.594, *P* = 0 ≤ 0.05, and the difference and the total effective rate are statistically significant. The direct impact of trust on reuse is 0.667, *P* = 0.033 ≤ 0.05, and the difference is statistically significant. The indirect impact of trust on reuse is 0.926, *P* = 0.04 ≤ 0.05, and the difference is statistically significant. Therefore, satisfaction plays a partial mediating role in the impact of trust on employee reuse after the merger.

## Discussion

M and As are widely used as an effective internationalization strategy given their associated synergies such as operational, economic, and financial synergies. Through the research, it has been found that the main intent for doing internationalization is to secure and share resources internationally to increase the level of competitiveness and profitability. Companies that internationalize or expand their business to other markets are likely to make good use of M and As as their internationalization strategy. Employee involvement is one of the main determinants of the effect of internationalization on organizational performance, and it refers to their involvement and commitment to internationalization. As for the factors that may hinder the effects of internalization, they include lack of adequate knowledge of the foreign market, lack of skills to appropriately analyze the foreign market, cultural differences, and miscalculation of statistics. As for cultural differences, the factors that create a downward shift in cultural management include geographical environment, historical background, and behavioral styles, and management patterns of the top management. The main factor that has been found to be important in the success of internationalization is the involvement of employees. It is important to the extent that even if an organization successfully implements its strategic vision, it still needs to depend on the ability of its employees to translate the vision into reality, and this can happen by obtaining and maintaining their support and commitment throughout the process of internalization. Therefore, organizations need to feel the value of recognizing the sense of recognition and expectations of employees during the process of business strategy formulation to be able to reap maximum benefits associated with internalization.

Employing a variety of tools, such as electronic questionnaires, Internet access, physical questionnaires, and interviews to collect anonymous data and to conduct model analysis, it is found that the main factors involved in the success of the process of internalization include employee involvement and consideration of different cultures. Both of these factors also play a considerable role in the success of M and As that lead to the improvement of organizational performance. The survey on the impact of internationalization shows that internationalization can improve organizational performance and profitability, but that if handled improperly, it will also hinder the organizational performance of some companies. The results of the impact of enterprise internationalization on performance are consistent with the research results in Sun et al. (2019). There is a positive correlation between the degree and scope of enterprise internationalization and performance. Table 3 displays the statistics of valid assumptions among the 14 companies.

**Table 3:** Statistics of valid hypotheses.

**Variable**		**Hypothesis**
Internal hypothesis	H1	The more co-workers motivate (use toolbox), the more they achieve job satisfaction.
	H2	Higher corporate culture (toolbox) influence leads to increased trust in the corporate culture.
	H3	Higher the ease of use leads to increased trust in corporate culture.
	H4	Higher degree of cultural trust of the (toolbox) corporation leads to more willingness to use and change the timing.
External hypothesis	H5	(Using toolbox) Higher performance expectation leads to higher cultural trust and job satisfaction.
	H6	The higher the learning (toolbox) expectation, the higher the job satisfaction.
	H8	(Using toolbox) Higher job satisfaction leads to increased willingness to continue to use the change.
	H9	Higher degree of corporate cultural trust (toolbox trust) leads to higher job satisfaction.
Moderator internal and external control	H10	The degree of trust in management culture (toolbox trust level) is stronger than the non-management level.
	H11	The degree of cultural trust (toolbox trust) of employees entering the corporation after the acquisition is stronger than that of the corporate employees.

As for the effects of the merger and acquisition, the results of the study show that prior to the merger, the total effect of trust on reuse was 0.264 and that the difference, as well as the overall effect of the merger and acquisition, was not statistically significant. This means that there was no correlation between trust and reuse. However, when the merger was done, the total effect of trust on reuse climbed up to 1.594, which means that the difference, as well as the total effect, was statistically significant. As for the figures for the direct effect of trust on reuse and the indirect effect of trust on reuse, the calculation and analysis of the data make it 0.667 and 0.926, respectively, which means that difference is statistically significant. This proves the role of satisfaction in the influence of trust on reuse once the merger and acquisition have been completed. In the research by Akiate, it is pointed out that the trust of employees in management greatly affects the fairness of management. Similarly, managerial equity also significantly affects all aspects of organizational commitment. Therefore, managerial equity acts as a partial mediator between dependent and independent variables, which is consistent with the results of this study.

## Conclusion

The purpose of this study is to explore the role of corporate culture accepted by employees in enterprise development and its impact on employees themselves. The influence of employee participation, cross-cultural management, and corporate culture on the enterprise is realized through the relevant literature. Then, investigation and analysis are carried out with American I Industrial Group as the research object to determine the impact of cross-cultural management on mergers and acquisitions and organizational performance. The results of the model experiment show that the total impact of trust on job satisfaction is 0.943, *P* = 0 < 0.05; therefore the difference is statistically significant, and the total effect is considered to be significant. Besides, the indirect impact of job satisfaction on trust is 0.432, *P* = 0 < 0.05; therefore, the difference is statistically significant. The direct impact of job satisfaction on reuse is 0.432, *P* = 0 < 0.05; therefore, the difference is statistically significant. Hence, for managers, job satisfaction plays a mediating role in the impact of trust on reuse. The job satisfaction of employees will increase when the mutual encouragement among colleagues increases. Corporate culture will affect the credibility of enterprises; the greater the role of corporate culture, the higher the credibility of the enterprise; the higher the credibility of the enterprise, the higher the employees' performance expectation, cultural trust, and job satisfaction; the higher the employee's learning expectation, the higher the job satisfaction; the higher the trust in the corporate culture, the higher the employee satisfaction. However, there are still some limitations. In cross-cultural teams, different teams have great institutional differences, and the object of this exploration cannot cover the whole industry. Therefore, in future research, more cross-cultural teams from different industries should be selected for research and analysis to make the conclusion more accurate.

## Data Availability Statement

The raw data supporting the conclusions of this article will be made available by the authors, without undue reservation.

## Ethics Statement

The studies involving human participants were reviewed and approved by Wenzhou University Ethics Committee. The patients/participants provided their written informed consent to participate in this study.

## Author Contributions

All authors listed have made a substantial, direct and intellectual contribution to the work, and approved it for publication.

## Conflict of Interest

The authors declare that the research was conducted in the absence of any commercial or financial relationships that could be construed as a potential conflict of interest.

## Publisher's Note

All claims expressed in this article are solely those of the authors and do not necessarily represent those of their affiliated organizations, or those of the publisher, the editors and the reviewers. Any product that may be evaluated in this article, or claim that may be made by its manufacturer, is not guaranteed or endorsed by the publisher.

## References

[B1] Aisyah F. Mursalin A. Octaviani D. Ekonomi F. (2020). Effect of corporate culture and work motivation of em- ployee performance of PT. pontianak harbor. J. Admin. Bus. Stud. 6, 14–19. 10.20474/jabs-6.4.1

[B2] Akiate Y. W. D. (2018). Employees' trust towards management and organizational commitment after a bank's merger and acquisition: mediated by procedural justice. Intern. J. Bus. Manag. Sci. 8, 12–15. 10.1108/IMR-02-2014-0046

[B3] Al-Louzi K. S. (2019). Factors impacting organizational innovation for managers: the case of jordanian public institutions. J. Bus. Manag. 7:2. 10.25255/jbm.2019.7.4.264.292

[B4] Almeida M. Coelho A. (2019). The antecedents of corporate reputation and image and their impacts on employee commitment and performance: the moderating role of CSR. Corp. Reput. Rev. 22, 10–25. 10.1057/s41299-018-0053-8

[B5] Alserhan H. Shbail M. (2020). The role of organizational commitment in the relationship between human resource management practices and competitive advantage in Jordanian private universities. Manag. Sci. Lett. 10, 3757–66. 10.5267/j.msl.2020.7.036

[B6] Dastgeer G. Rehman A. U. Asghar M. A. (2020). Selection and use of mediation testing methods; application in management sciences. Bus. Econ. Rev. 12, 1–26. 10.22547/BER/12.3.1

[B7] Faizaty N. E. Oktavia R. M. Mirza Dwinanda I. (2020). Implementasi organizational culture assessment instrument (Ocai) untuk pemetaan budaya organisasi Pt. Semen Indonesia (Persero) Tbk sebagai rujukan winning culture. Manajerial 7:37. 10.30587/manajerial.v7i01.1047

[B8] Farooq O. Farooq M. Reynaud E. (2019). Does employees' participation in decision making increase the level of corporate social and environmental sustainability? An investigation in South Asia. Sustainability. 11:511. 10.3390/su11020511

[B9] Frolova Y. Mahmood M. (2019). Variations in employee duty orientation: impact of personality, leadership styles and corporate culture. Eur. Bus. Rev. 9, 23–46. 10.1007/s40821-019-00135-8

[B10] Ili U. (2020). Measuring absenteeism as a precondition of quality management of absenteeism. Trendovi U Poslovanju 8, 66–74. 10.5937/trendpos2001066I

[B11] Kazi A. Chandani S. (2021). Local companies underperform: a comparative study of industries in Pakistan. Independ. J. Manag. Prod. 12, 1087–1106. 10.14807/ijmp.v12i4.1330

[B12] Kucharska W. Kowalczyk R. (2019). How to achieve sustainability-Employee's point of view on company's culture and CSR practice. Corp. Soc. Respons. Environ. Manag. 26, 453–467. 10.1002/csr.1696

[B13] Kussin L. Bundtzen H. (2021). How error prevention and organizational silence influences managers' self-perception - a repertory grid study. Bus. Ethics Leadership 5, 31–44. 10.21272/bel.5(1).31-44.2021

[B14] Najib H. Nawangsari L. C. (2021). Effect of intellectual capital on organizational sustainability with employee innovative behavior as intervening variables in Pt. Jaya maritime services. Eur. J. Bus. Manag. Res. 6, 158–163. 10.24018/ejbmr.2021.6.1.714

[B15] Odukoya J. A. Adekeye O. A. Agoha B. C. Olowookere E. Omonji D. O. (2020). The role of industrial-organizational psychology in sustainable development: implication for 21 st century employee management strategies. Test Eng. Manag. 82, 9857–9866.

[B16] Phan T. A. Nguyen T. T. K. Phan T. M. (2020). The S-shaped relationship between internationalization and performance: empirical evidence from laos. J. Asian Finan. Econ. Bus. 7, 357–366. 10.13106/jafeb.2020.vol7.no11.357

[B17] Rodríguez-Sánchez J. L. Mora-Valentín E. M. Ortiz-de-Urbina-Criado M. (2018). Successful human resources management factors in international mergers and acquisitions. Administ. Sci. 8:45. 10.3390/admsci8030045

[B18] Romani L. Barmeyer C. Primecz H. Pilhofer K. (2018). Cross-cultural management studies: state of the field in the four research paradigms. Intern. Stud. Manag. Org. 48, 247–263. 10.1080/00208825.2018.1480918

[B19] Schwens C. Zapkau F. B. Bierwerth M. Isidoe R. Knight G. A. Kabst R. (2018). International entrepreneurship: a meta-analysis on the internationalization and performance relationship. Entrepren. Theory Pract. 42, 734–768. 10.1177/1042258718795346

[B20] Shaheer N. Li S. Priem R. (2020). Revisiting location in a digital age: how can lead markets accelerate the internationalization of mobile apps. J. Intern. Mark. 28, 21–40. 10.1177/1069031X20949457

[B21] Smerek L. Vetráková M. (2020). Difference in human resources development in various types of companies. Polish J. Manag. Stud. 21, 398–411. 10.17512/pjms.2020.21.2.28

[B22] Song H. J. Yoon Y. N. Kang K. H. (2020). The relationship between board diversity and firm performance in the lodging industry: the moderating role of internationalization. Intern. J. Hosp. Manag. 86:102461. 10.1016/j.ijhm.2020.102461

[B23] Sosnilo A. I. Snetkova D. A. (2020). Employee health management as part of corporate culture and productivity tool. Admin. Consult. 2, 115–125. 10.22394/1726-1139-2020-9-115-125

[B24] Sun W. Price J. Ding Y. (2019). The longitudinal effects of internationalization on firm performance: the moderating role of marketing capability. J. Bus. Res. 95, 326–337. 10.1016/j.jbusres.2018.08.014

[B25] Svistunov V. Lobachyev V. Kuzina G. (2021). Organizational culture of Russian companies: state, problems and features of transformation. Manag. Pers. Intellect. Resou. Russia 9, 11–16. 10.12737/2305-7807-2021-11-16

[B26] Tran H. Q. Pham N. T. B. (2019). Organizational learning as a moderator of the effect of employee participation on academic results. Learn. Org. 26, 146–159. 10.1108/TLO-03-2018-0040

[B27] Trofimov A. Drobot O. Kokarieva A. Maksymova N. Lovochkina A. Kozytska I. V. (2019). The influence of management style and emotional intelligence on the formation of employees' commitment and loyalty. Hum. Soc. Sci. Rev. 7, 393–404. 10.18510/hssr.2019.7544

[B28] Urbancová U. Depoo L. (2021). Factors affecting strategic types of organizational culture: evidence from organizations and managers operating in the Czech Republic. Manag. Prod. Eng. Rev. 12, 48–59. 10.24425/mper.2021.136871

[B29] Uzyumova N. V. (2019). Adaptation of new employees as the criterion of social efficiency of company's corporate culture. Human. Soc. Sci. Bull. Finan. Univ. 9, 31–34. 10.26794/2226-7867-2019-9-3-31-34

[B30] Wang G. Zhang H. Xia B. Guangdong W. Han Y. Asce A. M. (2020). Relationship between internationalization and financial performance: evidence from ENR-Listed Chinese firms. J. Manag. Eng. 36:04019044. 10.1061/(ASCE)ME.1943-5479.0000736

[B31] Zhou Y. Fan X. Son J. (2019). How and when matter: exploring the interaction effects of high-performance work systems, employee participation, and human capital on organizational innovation. Hum. Resou. Manag. 58, 253–268. 10.1002/hrm.21950

